# Axiology and Ethical Responsibility in Pandemic Prevention: Value Conflicts, Disclosure, and Dialogical Public Health

**DOI:** 10.1192/j.eurpsy.2026.11890

**Published:** 2026-07-31

**Authors:** C. Lazzari

**Affiliations:** Psychiatry, International Centre for Healthcare and Medical Education (ICHME), London, United Kingdom

## Abstract

**Introduction:**

Pandemics intensify moral scrutiny of individual and collective behavior. Axiology—the philosophical study of values—offers critical insight into the ethical dilemmas surrounding disease prevention, personal autonomy, and social obligation. In pandemics such as HIV/AIDS and COVID-19, moral agency is illuminated through actions like mask-wearing, vaccination, and disclosure of health status, revealing tensions between rational knowledge and emotional reasoning.

**Objectives:**

To examine how axiological frameworks shape ethical behavior in pandemic contexts, particularly regarding prevention, disclosure, and interpersonal risk. The study explores how emotional ambivalence, stigma, and dialogical engagement influence public health adherence.

**Methods:**

A philosophical–clinical approach was employed, drawing on published case studies, historical analyses, and theoretical contributions from Douglas (2009), McClimans (2017), Thompson & Upshur (2017), and Lazzari et al. (1995–2016). Axiological principles were applied to assess the relational and moral dynamics embedded in disease-transmissible interactions, with emphasis on risk externalization, intimate vulnerability, and affective cognition.

**Results:**

Preventive ethics emerged as both procedural and emotional. Compliance with health guidance depended not solely on epistemic clarity but also on perceived fairness, dialogue, and trust. Concealment of health status illustrated the emotional cost of stigma and fear, while intimate relationships revealed moral conflict between desire and duty. Historical patterns of moral disengagement and affective denial significantly undermined prevention strategies.

**Table 1.** Table 1. long description.

Section	Key Concepts	Analytical Insights	Implications
**Results**	Dialogical ethics; emotional ambivalence; stigma; moral disengagement	Public health compliance shaped by perceived trust, fairness, and relational context	Affective dynamics and value conflicts influence behaviour beyond epistemic rationality
**Conclusions**	Axiological sensitivity; relational vulnerability; co-created prevention	Ethical behaviour during pandemics requires recognition of emotional and symbolic dimensions of risk	Calls for dialogical, community-engaged public health grounded in value pluralism and moral responsiveness

**Conclusions:**

Pandemic prevention must be framed not only through biomedical rationality but also through axiological sensitivity. Public health ethics requires attunement to emotional and relational contexts. Prevention is most effective when co-created with communities, acknowledging the value-laden dimensions of risk, responsibility, and care.

**Disclosure of Interest:**

None Declared

## Long descriptions

### Table 1. Long description

The table consists of four columns: Section, Key Concepts, Analytical Insights, and Implications. It contains two main rows of data.

Row 1: Results

- Key Concepts: Dialogical ethics, emotional ambivalence, stigma, and moral disengagement.

- Analytical Insights: Public health compliance is shaped by perceived trust, fairness, and relational context.

- Implications: Affective dynamics and value conflicts influence behavior beyond epistemic rationality.

Row 2: Conclusions

- Key Concepts: Axiological sensitivity, relational vulnerability, and co-created prevention.

- Analytical Insights: Ethical behavior during pandemics requires recognition of emotional and symbolic dimensions of risk.

- Implications: Calls for dialogical, community-engaged public health grounded in value pluralism and moral responsiveness.

Navigate back to Table 1..

